# Autologous myocutaneous flap implantation for chronic refractory chest wall sinus with infection: a case report

**DOI:** 10.1186/s13019-023-02205-5

**Published:** 2023-04-10

**Authors:** Lei Wang, Zhijun Liu, Zhongliang He, Chun Zhang

**Affiliations:** 1 Department of Cardiothoracic Surgery, Tongde Hospital of Zhejiang Province, 234 Gucui Rd., Hangzhou, 310012 Zhejiang China; 2 Department of Traumatology and Orthopedic Surgery, Tongde Hospital of Zhejiang Province, Hangzhou, 310012 Zhejiang China

**Keywords:** Myocutaneous flap, Chest wall sinus, Latissimus dorsi, Infection

## Abstract

**Background:**

Chest wall sinus with infection is a refractory disease caused by a variety of susceptible factors, and the treatment is still challenging. For clinically complex cases, although there are various surgical methods to choose from, it is still very difficult to achieve clinical cure, especially for patients with older age and many underlying diseases. Complete resection of chest wall sinus and application of repair and reconstruction technology may bring hope to refractory cases.

**Case presentation:**

Herein, we report a case of a 67 year-old woman who had undergone breast cancer surgery and a history of multiple cycles of radiotherapy and chemotherapy. One year ago, she had a fistula in the left chest wall with yellow purulent fluid. After admission to our hospital, chest computed tomography (CT) showed the formation of the left chest wall sinus, accompanied by high-density images of the left clavicle, part of the ribs and part of the sternu. According to the patient's symptoms, signs and imaging examination, we preliminarily diagnosed the patient as chest wall sinus with infection and chronic osteomyelitis. Therefore, in the first-stage operation, the patient underwent left chest wall sinus resection, left partial rib resection, left partial clavicular resection and left partial sternal resection, After surgery, the wound surface was changed with gauze dressing with sensitive antibiotic solution every day until the wound surface was clean and new granulation was formed. In the second-stage operation, the wound surface was appropriately expanded, and the pedicled latissimus dorsi myocutaneous flap was transferred to the chest wall defect. Finally, the skin paddle was sutured without tension to the normal skin around the chest, and two drainage tubes were placed. Anti-infection, anti-spasm, anti-coagulation and other treatments were given after operation, and the survival of myocutaneous flap, wound healing and sinus disappearance were observed.

**Conclusion:**

The application of pedicled latissimus dorsi myocutaneous flap in the treatment of intractable chronic chest wall sinus is an effective method. It does not change the shape of the thorax. The clinical effect is satisfactory in the near and medium term, which is worthy of clinical promotion.

## Introduction

The occurrence of chronic empyema and chest wall sinus is often caused by the presence of abscess cavity and sinus that cannot be completely eliminated after thoracic surgery, and may also be combined with bronchopleural fistula (BPF), residual bone and other necrotic tissues, making local infection difficult to control, and ultimately unhealed for a long time. It is always a challenge for thoracic surgeons to achieve complete cure [1–3]. For some cases of complex chest wall sinus with deep position or tortuous shape, it is difficult to achieve the curative effect only through dressing change or incision and drainage. Although with the continuous improvement of thoracic surgical techniques, there is still a certain incidence of chest wall sinus infection [4]. Due to long-term chronic infection, some patients with refractory chest wall sinus are often accompanied by chronic consumption or systemic poisoning symptoms, and even develop osteomyelitis, mediastinal infection and so on [5].

Chest wall sinus with infection is one of the common manifestations of chronic osteomyelitis. Due to the special position of chest wall bone and the important anatomical proximity, the treatment of chest wall sinus is quite difficult. In order to achieve the effect of clinical cure, it is necessary to ensure that there is no dead space and effusion in the tissue defect after debridement, thereby reducing the possibility of infection recurrence [6]. Although there are some clinical reports on the operation of the chest wall sinus with infection, there are few reports on the transfer of pedicled latissimus dorsi myocutaneous flap for the treatment of complex and huge chest wall tissue defects caused by chest wall sinus. It is critical not only to completely eliminate infectious lesion space, but also to keep chest wall shape as constant as possible [3].

Here we report a case of huge chest wall defect caused by complex chest wall sinus. It was successfully treated by transplantation of pedicled latissimus dorsi myocutaneous flap. The shape of chest wall remained basically unchanged after operation. There was no recurrence of chest wall sinus on chest CT.

## Case report

A 67 year-old female patient presented with left chest wall sinus one year ago. Local redness, pain and persistent purulent discharge occurred around the sinus orifice. Reviewing the medical history, the patient had undergone left mastectomy 20 years ago due to the diagnosis of left breast cancer, followed by regular radiotherapy and chemotherapy for several times. At the same time, she suffered from coronary heart disease, hypertension and other cardiovascular diseases. She underwent coronary stent implantation in 2017, 2019 and 2020 respectively. Currently, she regularly takes anticoagulant drugs (Aspirin Enteric-coated Tablets 100 mg QD) and antihypertensive drugs (Nifedipine Sustained Release Tablets 30 mg QD). Due to repeated non-healing of chest wall wound and formation of chest wall defect, ulcer and fistula caused by debridement, the patient was admitted to our hospital for further treatment. Chest CT scan showed partial defect of left chest wall with sinus formation, high-density shadow of left clavicle and some ribs. According to the patient's symptoms and imaging examination, we diagnosed chest wall sinus with infection and chronic osteomyelitis.

After admission, the patient continued to be given local cleaning and dressing changes on the wound surface. The purulent secretions at the sinus orifice were subjected to microbial culture and drug susceptibility tests. The results of the three secretion cultures were all suggestive of *Pseudomonas aeruginosa*. Her BMI was 18.4 kg/m^2^ and albumin was 24.5 g/L. We chose sensitive antibiotics for systemic anti-infective treatment. At the same time, nutritional support and correction of hypoalbuminemia were given. Enhanced chest CT and three-dimensional reconstruction were performed. Before chest CT examination, 50% meglumine solution was injected into sinus orifice through a thin drainage tube to fully understand the shape, scope and adjacent conditions of sinus. Due to the long course of disease and lack of confidence in treatment, the patients were given necessary psychological counseling and appropriate anti anxiety drug treatment.

**Fig. 1:**
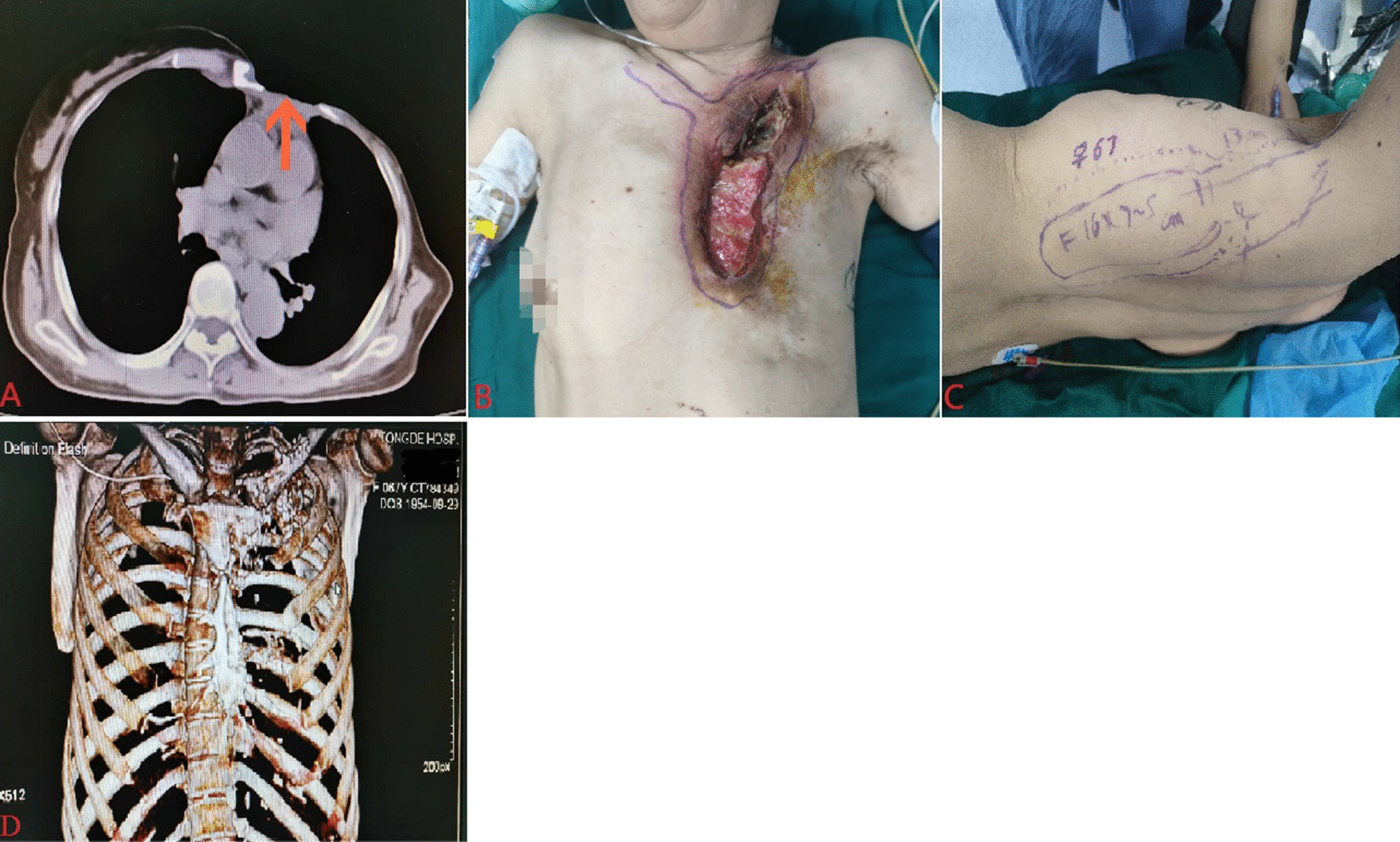
General information of patients before surgery. **A** Chest CT showed that the patient had a huge defect in the left anterior chest wall and part of the bone had been destroyed (Orange arrow). **B** After first-stage operation debridement of the left anterior chest wall sinus, the changes involved the sternum, rib and clavicle, and proliferative fresh granulation tissue was seen in some wound areas. **C** The range of pedicled latissimus dorsi myocutaneous flap was designed before operation. **D** Preoperative 3-D imaging showed high-density shadow and destruction of left clavicle, part of ribs and part of sternum

The operation was divided into two stages, both under general anesthesia. Before first-stage operation, we injected methylene blue solution from sinus orifice to make sinus wall fully stained to guide the scope of surgical curettage, which could not only ensure the complete removal of the diseased sinus wall tissue, but also avoided too much damage to the normal tissue and even the important organs behind the sternum. Taking the sinus orifice of chest wall as a center, a fusiform incision with a length of about 8 cm was made along the 1 cm around sinus orifice. The direction and length of the incision were determined according to the sinus shape (the sinus starts at the level of the left first rib and ends at the level of the left fourth rib, without communication with the thoracic cavity) shown on preoperative chest CT and the position of the myocutaneous flap to be filled. After incision of the skin, the sternum and infected area were fully exposed, sinus wall tissue was fully scraped with a curette, and then necrotic bone was completely removed with a rongeur (It starts from the junction of the left first rib and the manubrium cartilage. Part of the first rib, part of the lower edge of the left clavicle, and the adjacent medial end of the sternum are removed in sequence from near to far, from top to bottom, until the bone stump is fresh). When the chest wall wound tissue was fresh and there was no dye attached, we first rinse it with 1000 ml normal saline, then rinse it with diluted iodophor water (100 ml iodophor water and 400 ml normal saline) and 100 ml 3% hydrogen peroxide for one time, and then rinse it with 1000 ml normal saline again. Due to the huge wound defect after debridement, in order to ensure the cleanliness of the wound, improve local blood supply and prepare for the second-stage operation, vacuum sealing drainage (VSD) were performed after the operation (Fig. 1). We adjusted the pressure scale of the VSD device to 0.04 Mpa, and the device was removed after 7 days.

**Fig. 2:**
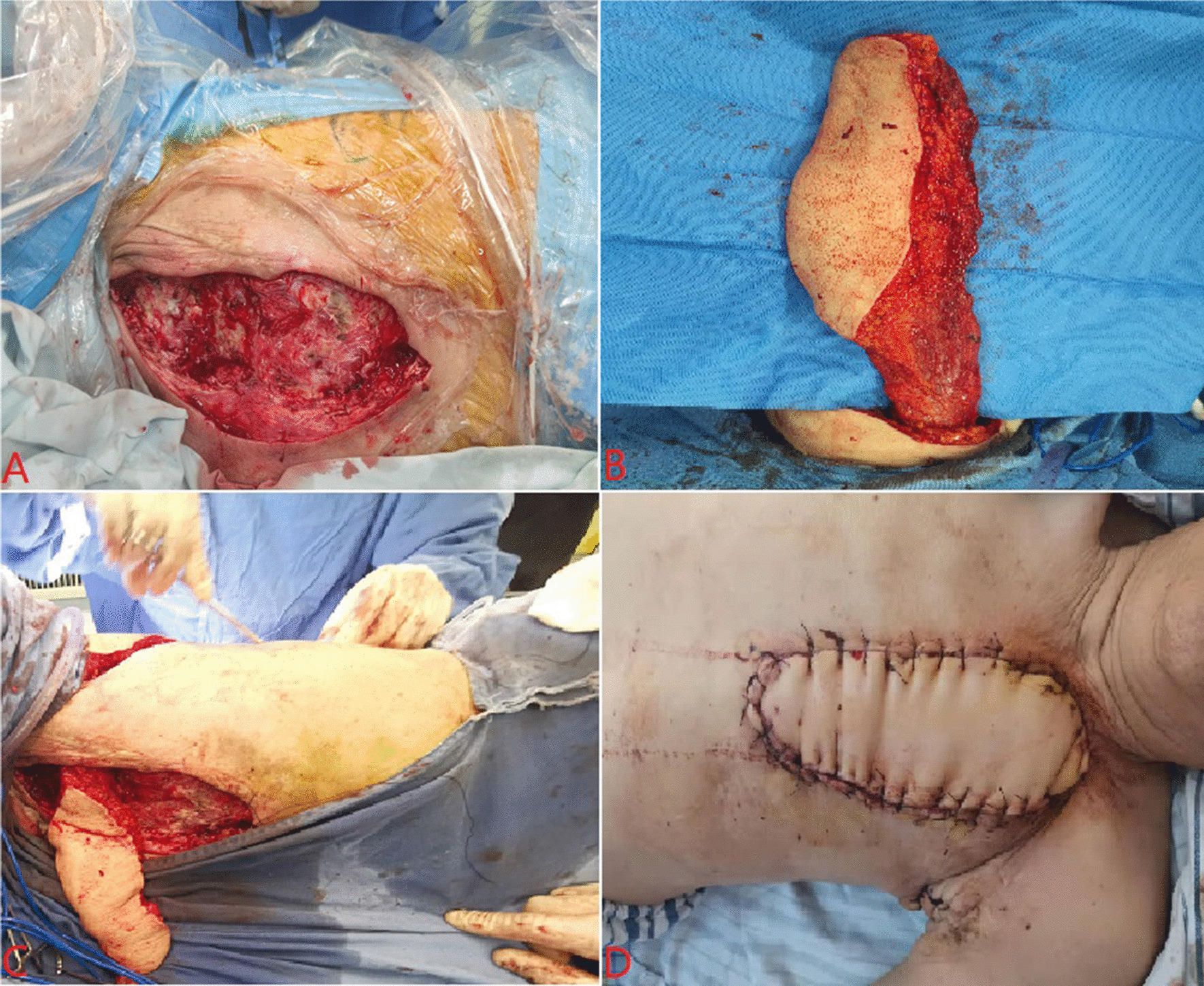
Intraoperative and postoperative general clinical data of patients. **A** During the second-stage operation, the sinus wound was debridement again. **B** A pedicled latissimus dorsi myocutaneous flap with a size of about 15 × 8 cm was harvested during the operation, and the blood supply was good. **C** The latissimus dorsi myocutaneous flap was transferred to the chest wall defect by opening the subcutaneous tunnel. **D** On the 4th day after operation, the chest wall defect was repaired, the drainage tubes were pulled out, and the myocutaneous flap survived

**Fig. 3:**
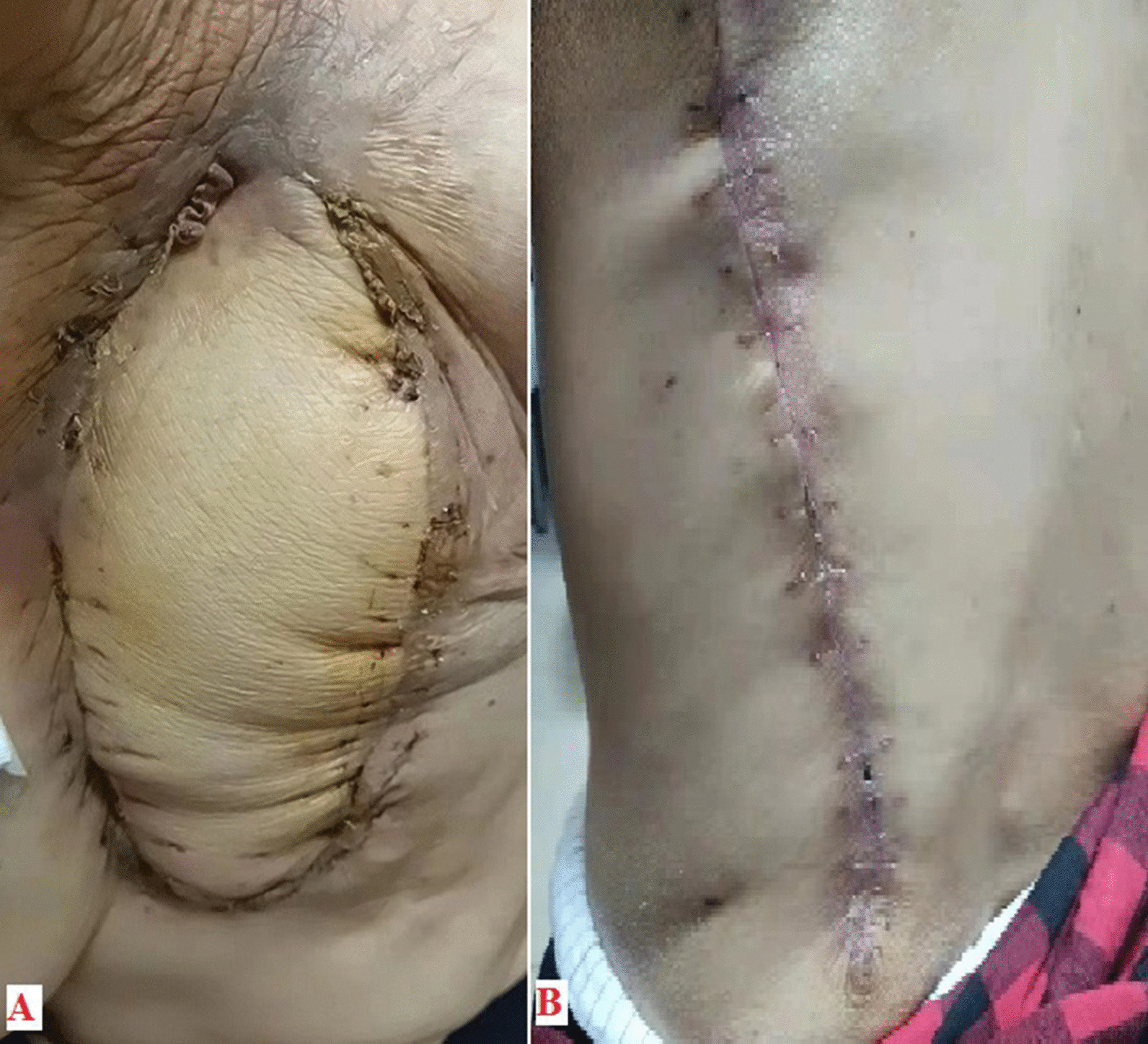
Postoperative incision images 6 months after surgery. **A** The defect of chest wall disappeared and the myocutaneous flap fully survived. **B** The postoperative incision on the back healed well

When the patient's general condition was good after first-stage operation, after the vacuum sealing drainage device was removed, the daily incision dressing change, anti-infection, nutritional support and other treatments were continued. When the granulation of sinus wound was fresh, the secretion was significantly reduced, the re-culturing of the wound secretion was negative, and the infection was preliminarily controlled, we were ready for the second-stage operation. Continue to appropriately expanded the wound along the original incision, thoroughly debrideed the necrotic tissue until the wound was fresh, and measured the size of the chest wall defect was about 15 × 8 cm. After hemostasis, rinsed the wound with a large amount of iodophor, hydrogen peroxide, and normal saline alternately. The patient's body position was changed to 90° lateral lying position. According to the preoperative planned flap incision, the pedicled latissimus dorsi myocutaneous flap was selected as the living tissue to fill the defect wound. The computed tomography angiography (CTA) examination of the subclavian artery showed that there were internal thoracic artery and thoracodorsal artery on the affected side without malformation. During the operation, first of all, an incision was made along the outer edge of latissimus dorsi muscle, starting from the proximal axillary apex, and the proximal and distal ends of latissimus dorsi muscle were dissociated and fully exposed in turn to protect the thoracodorsal artery and vein. Finally, a latissimus dorsi myocutaneous flap with thoracodorsal neurovascular bundle of sufficient length was formed. The size of the latissimus dorsi myocutaneous flap was about 30 × 8 cm, while the skin island of about 16cmx8cm was reserved. During harvesting of the myocutaneous flap, the blood supply should be preserved as much as possible, while avoiding excessive distortion of the pedicle of the myocutaneous flap. We first made a subcutaneous tunnel between the acquisition site of the myocutaneous flap and the sternal wound, transferred the myocutaneous flap to the chest wall defect wound, then sutured and fixed it with the soft tissue around the wound, closely combined the myocutaneous flap with the bottom of the chest wall wound to eliminate the dead space, and finally placed a drainage tube (Fig. 2). The patient was treated with anti infection (Cefoperazone Sodium and Sulbactam Sodium + Levofloxacin and Sodium Chloride Injection), anti spasm (Raceanisodamine Hydrochloride Injection) and anticoagulation (Enoxaparin Sodium Injection) after operation. The color and temperature of the myocutaneous flap was closely observed and tested. The transplanted myocutaneous flap was kept warm, and the incision was avoided from compression to protect the smooth blood flow. The patient pulled out the chest tubes on the 4th postoperative day and was discharged from the hospital on the 7th day. At present, the patient was followed up for 6 months after the operation, the incision healed well (Fig. 3) and no malignant tumor tissue was found in the routine pathology after the two operations. Repeat chest CT showed that the chest wall sinus disappeared completely, and the pedicled myocutaneous flap fully survived.

## Discussion and conclusion

The complex chest wall sinus can reach deep into the sternum from the body surface and spread to the ribs and even clavicle. It often presents a pathological blind lumen, while the morphology of the lumen is complex and diverse. The lumen wall is full of unhealthy necrotic bone and granulation tissue, often accompanied by pathogenic microbial infection [7]. Chest wall sinus is often caused by chest infection, trauma or postoperative radiotherapy and chemotherapy of chest and neck tumors. Chronic sinus wall fibrous organic thickening, accompanied by skin scar formation, and infection caused by multiple branches of sinus is difficult to control. *Pseudomonas aeruginosa* had the highest detection rate in surgical trauma infection. Its pathogenic substances are mainly endotoxin, exotoxin and proteolytic enzymes. Its pathogenic characteristic is to cause secondary infection, which mostly occurs when the body's immunity is reduced [8]. Therefore, we need to design a surgical method that can completely solve these problems and achieve one-stage cure [9].

The patient has a clear history, localized swelling and sinus formation have occurred at the time of treatment, and a small amount of purulent secretion can be seen flowing out of the sinus orifice. Its clinical diagnosis is not difficult. We choose to inject meglumine diatrizoate solution from the sinus orifice before chest CT examination, that is, contrast fistulography, which can show the shape, depth and branches of the sinus to a certain extent, so as to provide basis for the formulation of operation plan. Because the shape of sinus is often tortuous, complex and changeable, ordinary dressing change, disinfection and debridement are difficult to go deep into the interior of the sinus, so the complex chest wall sinus often discharges pus repeatedly, and the patient is in great pain. In the process of diagnosis and treatment, although thorough debridement can alleviate the progress of the disease to a certain extent, the huge chest wall defect is the key to follow-up treatment, especially the repair and reconstruction in an ideal state.

The reconstruction of chest wall mainly includes bone reconstruction and soft tissue reconstruction. Bone reconstruction generally refers to the use of hard materials to reconstruct bone defects and restore the integrity of the thorax. Bone chest wall reconstruction materials mainly include autologous tissue materials, biological materials and artificial materials. Titanium alloy products developed in recent years have high hardness, low density, easy to shape, stable physical and chemical properties and good tissue compatibility, which do not affect postoperative magnetic resonance imaging (MRI) examination, and gradually become one of the most common repair materials. The application of 3D printing technology in chest wall reconstruction is also more precise and individualized [10–14]. Soft tissue repair mainly relies on the transfer of soft tissue flap to complete the tightness and aesthetics of the chest wall, commonly including latissimus dorsi muscle flap, pectoralis major muscle flap, rectus abdominis muscle flap, greater omentum flap and free myocutaneous flap [15]. Autogenous material is the first choice for chest wall soft tissue reconstruction because of its convenience, no rejection reaction and stable and reliable reconstruction effect. Since most of the reconstruction operations do not involve the organs in the pleural cavity, only part of the parietal pleura is removed, which can rely on mucosal crawling or pseudomembrane formation without parietal pleura repair. When necessary, muscle flap, fascia, greater omentum flap and biomaterial can also be used to cover important organs or their stumps [16, 17].

For various sinus tissues caused by radiotherapy of malignant tumors in the body, the principle of treatment is early debridement and adequate drainage. In order to avoid the invalid cavity formed by deep cavity wound after debridement, various surgical methods are often needed to repair the wound. The wounds with shallow location and small defect area can be repaired by local flap transfer. On the contrary, using pedicled myocutaneous flap or free myocutaneous flap is a common method. When the pedicled myocutaneous flap near the diseased tissue cannot be repaired or fails to repair, the distal free myocutaneous flap can be reconstructed using microsurgical techniques [18, 19]. The tissue flap transplantation carried out in recent years can not only eliminate the residual cavity and sinus, but also keep the shape of the thorax basically unchanged. Moreover, the free myocutaneous flap does not need skin grafting due to the huge skin defect of the chest wall. After operation, the color of the skin paddle can be observed regularly and the blood supply can be understood in time. Whether it is the chest wall defect caused by chest tumor or chronic infection, the application of autologous tissue flap for chest wall reconstruction has become more and more widespread [20–22]. In order to eliminate the chest wall tissue defect caused by debridement, increase local blood supply, improve local tissue healing and anti infection ability are very important. For patients with large tissue defect after debridement, pedicled myocutaneous flap with good blood supply should be used to fill the chest wall defect [23, 24]. Since the patient has undergone total mastectomy for breast cancer, and the nearby pectoralis major muscle and surrounding tissues are weak, we chose the latissimus dorsi myocutaneous flap with close anatomical location and abundant blood supply to repair the huge chest wall defect [25, 26].

For the case with large chest wall defect and history of breast cancer surgery, it is a good scheme to transfer the pedicled latissimus dorsi myocutaneous flap to fill the defect wound by subcutaneous tunnel. The advantages are as follows: (a) it is easy to anatomy, easy to obtain, large in volume and rich in muscle tissue, which can meet the filling requirements of nearby huge chest wall defects; (b) Generally, there will be no serious complications. It can be sutured in one stage without skin grafting; (c) The pedicled myocutaneous flap has rich blood supply and strong anti-infection ability. It has unique advantages for fixed chest wall residual cavity infection; (d) Due to its strong plasticity, fully free pedicled myocutaneous flap can fully fill the residual cavity and its dead corner as long as its width and dimension are sufficient; (e) The thoracic function and stability are less affected, and the preserved skin paddle is convenient to observe the blood supply after operation; (f) Due to the strong activity of the musculocutaneous flap, it is easy to activate and repair the connective tissue with the chest wall wound, so as to easily generate a new vascular network and further promote the dissipation of inflammation; (g) The success rate of operation is high. Generally, secondary operation will not be performed due to poor blood supply. The operation time is short and the incidence of complications such as thrombosis is low [27–30]. According to the postoperative follow-up observation of this patient, the myocutaneous flap fully survived and the quality of life was significantly improved.

To sum up, it can be seen from the diagnosis and treatment process of this case that although the huge chest wall defect caused by chronic chest wall sinus is rare, it is harmful. At the same time, the patient has experienced total mastectomy. The patients are older and have many basic diseases. In order to avoid the fatal consequences caused by the in-depth progress of sinus, surgery should be performed as soon as possible on the basis of actively improving the general condition. From the short-term follow-up of patients, the application of pedicled latissimus dorsi myocutaneous flap in the treatment of huge chest wall defect caused by complex chest wall sinus is an effective method, and the design of myocutaneous flap is flexible. It is easy to cut and keep the shape of the thorax unchanged, and the clinical effect is satisfactory.

## Data Availability

The datasets used and/or analysed during the current study are available from the corresponding author on reasonable request.

## Abbreviations

CT: Computed tomography
BPF: Bronchopleural fistula
VSD: Vacuum sealing drainage
CTA: Computed tomography angiography
MRI: Magnetic resonance imaging

## References

[1] Walsh MD, Bruno AD, Onaitis MW (2011). The role of intrathoracic free flaps for chronic empyema. Ann Thorac Surg.

[2] Deshpande M, Kamath A, Allinson K (2011). A 76-year-old lady with chronic cough and a discharging chest wall sinus. Thorax.

[3] Xiaowen H, Zhongliang H, Lifeng S (2018). Free musculocutaneous flap transfer for refractory chronic empyema with chest wall sinus in a 43-year-old male with hemophilia A. J Thorac Dis.

[4] Zhongliang H, Lifeng S, Weihua X (2021). Treatment of the postoperative refractory empyema with a bronchopleural fistual by a pedicled or free muscle flap transplantation. Chin J Plast Surg.

[5] Zhongliang H, Lifeng S, Weihua X (2020). Effective treatment of bronchopleural fistula with empyema by pedicled latissimus dorsi muscle flap transfer: two case report. Medicine.

[6] Hongwei G, Weisheng K, Yiquan L (2018). Clinical analysis of chronic sternal osteomyelitis with sinus tract after cardiovascular surgery. Natl Med J China.

[7] Hauser J, Steinau HU, Ring A (2014). Sternal osteomyelitis. Etiology, diagnosties and operative therapy concepts. Chirurg.

[8] Dantis K, Kumar Dewan R (2022). Surgical outcomes and the factors affecting lung expansion following open window thoracostomy in chronic tuberculous empyema with destroyed lung. Asian Cardiovasc Thorac Ann.

[9] Yongyong W, Zhongliang H, Chun Z (2021). Free vastus lateralis muscle flap transplantation for postoperative chronic empyema: retrospective analysis of eight case series. Ann Palliat Med.

[10] Bongiolatti S, Voltolini L, Borgianni S (2017). Short and long-term results of sternectomy for sternal tumours. J Thorac Dis.

[11] Losken A, Thourani VH, Carlson GW (2004). A reconstructive algorithm for plastic surgery following extensive chest wall resection. Br J Plast Surg.

[12] Naoum GE, Salama L, Ho A (2019). The impact of chest wall boost on reconstruction complications and local control in patients treated for breast cancer. Int J Radiat Oncol Biol Phys.

[13] Zhang Y, Li JZ, Hao YJ (2015). Sternal tumor resection and reconstruction with titanium mesh: a preliminary study. Orthop Surg.

[14] Wang L, Cao T, Li X (2016). Three-dimensional printing titanium ribs for complex reconstruction after extensive posterolateral chest wall resection in lung cancer. J Thorac Cardiovasc Surg.

[15] Russell RC, Feller AM, Elliott LF (1991). The extended pectoralis major myocutaneous flap: uses and indications. Plast Reconstr Surg.

[16] Moradiellos J, Amor S, Córdoba M (2017). Functional chest wall reconstruction with a biomechanical three-dimensionally printed implant. Ann Thorac Surg.

[17] Scarnecchia E, Liparulo V, Pica A (2017). Multidisciplinary approach to chest wall resection and reconstruction for chest wall tumors, a single center experience. J Thorac Dis.

[18] Delanian S, Lefaix JL (2007). Current management for late normal tissue injury: radiation-induced fibrosis and necrosis. Semin Radiat Oncol.

[19] Martinez DC, Badhey A, Cervenka B, Zender C, Tang A, Patil Y (2020). Surgical techniques for head and neck reconstruction in the vessel-depleted neck. Facial Plast Surg.

[20] Sakuraba M, Umezawa H, Miyamoto S (2017). Reconstructive surgery for bronchopleural fistula and empyema: new application of free fascial patch graft combined with free flap. Plast Reconstr Surg Glob Open.

[21] Matros E, Disa JJ (2011). Uncommon flaps for chest wall reconstruction. Semin Plast Surg.

[22] Luan A, Galvez MG, Lee GK (2016). Flow-through omental flap to free anterolateral thigh flap for complex chest wall reconstruction: Case report and review of the literature. Microsurgery.

[23] Zhongliang H, Lifeng S, Weihua X (2021). An 83-year-old-male with bronchopleural fistula and empyema successfully treated with multidisciplinary management of thoracostomy, endoscopic, and surgical treatment: a case report. Ann Transl Med.

[24] Ried M, Gies S, Potzger T (2016). Surgical reconstructive procedures of the chest wall after mediastinitis. Chirurg.

[25] Shamji FM (2020). Sepsis in the postpneumonectomy space: pathogenesis, recognition, and management. Thorac Surg Clin.

[26] Kim WJ, Kim WS, Kim HK (2018). Reconstruction of small chest wall defects caused by tubercular abscesses using two different flaps. Ann Thorac Surg.

[27] De Palma A, Maruccia M, Di Gennaro F (2020). Right thoracotomy approach for treatment of left bronchopleural fistula after pneumonectomy for tubercolosis. General Thorac Cardiovasc Surg.

[28] Moyano-Bueno D, Blanco JF, López-Bernus A (2019). Cold abscess of the chest wall: a diagnostic challenge. Int J Infect Dis.

[29] Iga N, Fuchimoto Y, Koyanagi T (2021). A rare case of chest wall tuberculosis: tuberculous scapulothoracic bursitis. Respir Med Case Rep.

[30] Kabiri EH, Alassane EA, Kamdem MK (2020). Tuberculous cold abscess of the chest wall: a clinical and surgical experience. Report of 16 cases (case series). Ann Med Surg (Lond).

